# The relationship between childhood maltreatment and social anxiety among Chinese male individuals with drug use disorders: a moderated mediation model of fear of negative evaluation and self-construals

**DOI:** 10.3389/fpsyg.2023.1193952

**Published:** 2023-11-27

**Authors:** Yang Liu, Hao Zhang, Hualing Miao, Jia Zhang, Cheng Guo

**Affiliations:** ^1^Research Center of Mental Health Education, Faculty of Psychology, Southwest University, Chongqing, China; ^2^School of Education and Psychology, Chengdu Normal University, Chengdu, China

**Keywords:** childhood maltreatment, fear of negative evaluation, self-construals, social anxiety, Chinese male individuals with drug use disorder

## Abstract

**Background:**

Social anxiety (SA) is prevalent among individuals with drug use disorders, playing a significant role in the etiology and maintenance of drug addiction. The etiological model of SA suggests a link between the development of SA and childhood maltreatment. Childhood maltreatment not only acts as a complex trauma with negative effects on individuals’ selves and other cognitions but also exerts a negative influence through early negative parent–child interactions on individuals’ internal working models, leading to the development of fear of negative evaluation and SA. Furthermore, self-construals, as a personality trait that emerges from the framework of the theory of sociocultural models, may exert a moderating effect on these mechanisms. The present study utilized a moderated mediation model to examine how childhood maltreatment relates to SA in individuals with drug addiction, aiming to provide support for a comprehensive understanding and effective resolution of SA in this group.

**Methods:**

A total of 618 Chinese male individuals with drug addiction (M = 34.13, SD = 8.76) participated, and they completed the Childhood Trauma Questionnaire Short Form, the Fear of Negative Evaluation Scale, the Self-Consciousness Scale’s Social Anxiety Subscale, and the Self-Construal Scale. SPSS PROCESS Macro was used to analyze the data.

**Result:**

Correlation analysis revealed weak correlations among all variables but strong correlations between the SCS subscales. Mediation analyses revealed that fear of negative evaluation partially mediated the association between childhood maltreatment and SA. Moderated mediation analyses revealed that the link between fear of negative evaluation and SA was moderated by independent self-construal. The association was stronger among those with high independent self-construal than among those with low independent self-construal. An integrative moderated mediation analysis indicated that independent self-construal positively moderated the indirect association between childhood maltreatment and SA via fear of negative evaluation. However, interdependent self-construal did not show a moderated effect.

**Conclusion:**

Fear of negative evaluation plays a partial mediating role in the relationship between childhood maltreatment and SA, while independent self-construal enhances the association between fear of negative evaluation and SA. Decreasing the fear of negative evaluation and intervening in self-construals may attenuate the association between childhood maltreatment and SA among Chinese male individuals with drug addiction.

## 1 Introduction

Social anxiety (SA) is a negative emotion that individuals experience in real or imaginary social interaction situations due to the fear or apprehension of receiving negative evaluations from others (Zhao et al., 2020). When this emotion reaches a pinnacle of severity such that functioning is impaired, we refer to it as social anxiety disorder (SAD) or social phobia (Morrison and Heimberg, 2013). The SA theoretical model proposed by Heimberg et al. (2010) highlights the significant role of adverse social experiences and/or distorted self-perceptions in maintaining SA within potential social evaluation environments. More specifically, the negative self-image resulting from adverse social experiences and/or distorted self-perceptions leads socially anxious individuals to doubt their ability to meet high standards in social situations. The perceived likelihood of negative evaluation from the audience and the undesirable social consequences that follow from it (e.g., rejection, loss of status) is likely to be high. Anticipation of negative evaluation elicits anxiety and the devaluation of the mental representation of the self as seen by others, creating a maladaptive negative feedback loop. Data from the NCS indicates that the overall lifetime prevalence of SA disorder in the general population is 13.3%, with a rate of 15.5% for female people and 11.1% for male people (Kessler et al., 1994). In a meta-analysis conducted by Tang et al. (2022), it was found that the prevalence of SA disorder among children, adolescents, and young adults in China is 2.1%, while the prevalence of SA symptoms is 23.5%. Multiple findings indicate a prevalent occurrence of SA among individuals with drug use disorders. For example, Grenyer et al. (1992) reported that approximately 25% of a sample of opioid users experienced SA. Buckner et al. (2016) demonstrated that 31.7% of cannabis users were diagnosed with SA disorder. Shams Eldin et al. (2019) conducted a study on anxiety disorders in patients recovering from drug dependence and found that 18% of the individuals in the recovered group had SA disorder. The National Comorbidity Study (NCS) revealed a substantial comorbidity between drug use and SA disorder, with nearly one-third to one-fourth of individuals with cannabis dependence also having SA disorder (Agosti et al., 2002).

Based on the above data, it is evident that individuals with drug addiction exhibit higher levels of SA than the general population. While there is currently no precise data regarding SA or SAD among Chinese individuals with drug use disorders, it is our belief that the levels of SA may be high among them. Existing research has shown that the prevalence of SA in East Asian countries is higher than in Western nations (Norasakkunkit and Kalick, 2009; Woody et al., 2015). Surveys indicate that Chinese university students exhibit significantly higher levels of SA compared to American norms (Ling et al., 2005). The underlying reasons behind these research findings might be attributed to the relatively strong emphasis on interpersonal harmony in collectivist cultures (Han and Humphreys, 2016), which places higher demands on individual social interactions. Hence, the negative social image of individuals with substance use disorders exacerbates their interpersonal challenges within a collectivist cultural context. Survey research indicates that Chinese individuals with drug use disorders commonly experience social prejudice, workplace discrimination, and family misunderstanding (Yan, 2019), which contribute to the high prevalence of SA among this population. Furthermore, individuals with drug use disorders exhibit impaired executive functioning, which may lead to difficulties in social interactions and result in emotional distress (Lewis et al., 2009). At the same time, executive function deficits can also give rise to challenges in emotion regulation, limiting an individual’s ability to regulate negative emotions, thus potentially leading to more severe SA (Formiga et al., 2021). Research has demonstrated that male people tend to display higher levels of implicit negative emotion than explicit expression (Lan and Zhang, 2015). This disparity in emotional expression partly reflects male coping mechanisms, characterized by emotional repression, which may intensify psychological conflicts and hinder the resolution of negative emotions such as SA. Buckner et al. (2012) confirmed that SA plays a significant role in the etiology and maintenance of drug addiction, and this effect may be more pronounced in the male population. A study conducted on community adolescents has provided evidence that relative to female people, male people exhibit a stronger relationship between SA and their substance use (Wu et al., 2010). In conclusion, SA exhibits higher prevalence and adverse consequences among Chinese male individuals with drug use disorders. To gain a comprehensive understanding and effectively address SA among Chinese male individuals with drug use disorders, it is crucial to investigate the underlying mechanisms involved in their experience of SA.

Childhood maltreatment (CM) serves as a significant influencing factor in the emergence of numerous psychological issues (DiLillo et al., 2007; Aff et al., 2012; Kong et al., 2019), of which SA is one. According to the World Health Organization (WHO), CM can be defined as “all forms of physical and/or emotional ill-treatment, sexual abuse, neglect or negligent treatment, or commercial or other exploitation, resulting in actual or potential harm to the child’s health, survival, development, or dignity in the context of a relationship of responsibility, trust, or power,” and the WHO categorizes CM into five main types: childhood physical abuse, childhood sexual abuse, childhood emotional abuse, childhood physical neglect, and childhood emotional neglect (WHO, 2016). A review of the environmental risk factors for SA has proven that maladaptive parenting and early traumatic events are associated with the development of SA (Brook and Schmidt, 2008). Additionally, etiological models of SA disorder propose a link between the development of SA and CM (Kuo et al., 2011), a widely recognized global public health issue frequently observed among individuals with drug use disorders. Grice et al. (1995) reported that more than 60% of individuals with drug addiction experienced physical and/or sexual abuse during their childhood. A meta-analysis study on the prevalence of CM in individuals with opioid use disorder (OUD) revealed that the prevalence of sexual abuse was 41% among women and 16% among men. Among all individuals with OUD, prevalence estimates were 38% for physical abuse, 43% for emotional abuse, 38% for physical neglect, and 42% for emotional neglect (Santo et al., 2021). Khoury et al. (2010) found that 34% of individuals with cocaine dependence reported a history of physical and/or sexual abuse. Comparatively, the detection rate of CM is lower in the general population. A comprehensive review study on CM among the general population worldwide revealed that nearly one-quarter of adults globally (22.6%) experienced physical abuse during childhood, 36.3% endured emotional abuse, and 16.3% experienced physical neglect. Gender differences were observed in childhood sexual abuse, with a prevalence of 18% among girls and 7.6% among boys (Abbasi et al., 2015). Importantly, CM exposes individuals to long-term negative interpersonal experiences during a critical period of growth. These experiences can influence the development of internal working models that guide social interactions in adulthood (Bretherton and Munholland, 1999; Feeny et al., 2008). Attachment theory considers that the internal working models built by early parent–child interactions influence the characteristics of individual lifelong interpersonal adaptation. Griffin and Bartholomew (1994) defined two underlying dimensions of internal working models: the self-model and the others model. By considering the positivity and negativity of each dimension, four distinct prototypes can be identified. Individuals who have experienced CM may develop negative internal working models of both the self and others. Consequently, they are more likely to exhibit heightened attention to negative information in interpersonal environments, underestimate their own self-image (Reyes, 2008), and overestimate the potential threat posed by others during social interactions (Huh et al., 2017), which leads to fear of negative evaluation and associated SA.

At the same time, due to its typically chronic nature and its cumulative impact on psychological adjustment, CM is considered a complex trauma (Courtois, 2004), which contributes to the risk of developing complex post-traumatic stress disorder (CPTSD; Herman, 2012; Cloitre et al., 2019; Ford and Courtois, 2021). The CM environment fails to facilitate the development of age-appropriate adaptive coping strategies for dealing with stress and negative emotions in children. Instead, children often rely on negative coping strategies such as aggression, dissociation, and avoidance to manage the stress and extreme emotions resulting from maltreatment (Cicchetti and Valentino, 2006). While short-term negative coping may serve a self-protective function, excessive reliance on these strategies hinders the processing and integration of memories and experiences. This, in turn, is believed to lead to a fragmented or distorted self and other understandings, forming the basis for many symptoms related to complex trauma (Bailey et al., 2007). Courtois (2004) summarized seven consequences of complex trauma, among which three may serve as significant foundations associated with the link between CM and SA: emotional dysregulation, negative self-concept, and disturbances in relationships. These factors constitute essential components of the core symptoms of ICD-11 complex post-traumatic stress disorder (CPTSD), distinguishing CPTSD from PTSD (Maercker, 2021), and they play a significant role in the emergence of SA. A study on CM and suicidal ideation found an association between CM and negative emotions such as guilt and shame. These dysregulated negative emotions, which reflect the identification of the abused individuals with their abusers (Sekowski et al., 2020), play a significant role in the emergence of SA (Cândea and Szentagotai-Tătar, 2018). Penner et al. (2019) conducted research that confirmed that CM affects identity integration during adolescence, resulting in identity diffusion. This is believed to lead to chronic issues in social relationships (Beeney et al., 2015). In recent years, researchers have begun to focus on the development of the sense of self in individuals with substance use disorder (SUD) and its relationship with early adverse experiences. Wojtynkiewicz et al. (2021) confirmed that individuals with SUD exhibited lower levels of self-identity than the control group. Furthermore, a study conducted on individuals with SUD demonstrated a negative association between insecure attachment and individual self-identity (Wojtynkiewicz and Sekowski, 2022). This indicates that substance-dependent individuals who have experienced CM struggle to form a healthy self-identity, which affects the stability and consistency of their self-awareness, subsequently influencing the formation and shaping of self-concept and rendering their social interactions susceptible to negative influences, thus contributing to the emergence of SA. At the same time, fear of negative evaluation is a typical variable reflecting negative self-image in interpersonal situations (Ng et al., 2014), and it not only is influenced by early negative interpersonal experiences (Zlomke et al., 2016) but also constitutes a cognitive component of SA (Heimberg et al., 2010). Moreover, research has confirmed that different self-construals produced under the influence of an acquired sociocultural environment make individuals have different attitudes and cognitions toward others and the environment to which they belong (Lam, 2006). This factor influences individuals’ perceptions of themselves and others within interpersonal situations and their related social emotions. Overall, this study is designed to assess the mediating role of fear of negative evaluation and the moderating role of self-construals in the association between CM and SA among Chinese male individuals with drug use disorders.

### 1.1 The mediating role of fear of negative evaluation

Fear of negative evaluation is a typical issue of social communication that manifests as apprehension about others’ evaluations, distress over their negative evaluations, avoidance of evaluative situations, and the anticipation that others would evaluate oneself negatively (Watson and Friend, 1969; Collins et al., 2005; Ng et al., 2014). The cognitive model of SA posits that the fear of negative evaluation serves as an important integral component of the cognitive aspects of SA (Weeks et al., 2008; Heimberg et al., 2010). According to this model, the core fear that drives SA is the negative perception of oneself, described as “characteristics of the self that one perceives as being deficient or contrary to perceived societal expectations.” It is the key to triggering fear of negative evaluation and associated SA (Moscovitch, 2009).

The looking-glass self-theory implies that people shape their self-concept based on their understanding of how others perceive them (Oikawa and Yoshida, 2007). People with a CM history develop negative internal working models, in turn leading to the formation of a negative self-view because they lose necessary positive support from others and are treated negatively by others (Speidel et al., 2023). At the same time, children learn their self-worth from the reactions of others, especially caregivers. CM as complex trauma leads to alterations in self-perception and difficulties with self-concept (Herman, 2015). Studies have found a significant positive correlation between CM and negative views of the self (Devi et al., 2013; Flynn et al., 2014), which accounts for the association between CM and fear of negative evaluation. Therefore, CM may be a predictive factor of fear of negative evaluation. Researchers have started investigating the mediating effect of fear of negative evaluation and related concepts on the relationship between CM and affective problems. Lucero et al. (2022) found that fear of negative evaluation mediates the association between childhood trauma and affective disorders. Garety et al. (2001) and Morrison et al. (2003) proposed a cognitive model of affective problems or disorders that suggests that childhood adverse experiences may lead to a cognitive vulnerability of affective problems characterized by negative self and environmental evaluations. As a result, CM contributes to the development of fear of negative evaluation, which, in turn, leads to SA among Chinese male individuals with drug use disorders. Therefore, we propose the following hypothesis:

*Hypothesis 1*: Fear of negative evaluation may play a mediating role in the relationship between CM and SA.

### 1.2 The moderating role of self-construals

The theory of sociocultural models (TSCM) considers that the sociocultural environment of which individuals are a part plays an important role in influencing individuals’ cognitions, motivations, emotions, selves, and other mental capacities (Chirkov, 2020). Self-construal is a personality characteristic that arises in this theoretical context. It is a significant cultural feature that influences human behavior and explains cultural differences in behavior, cognition, and emotion (Han and Humphreys, 2016). Furthermore, self-construal is conceptualized as a constellation of thoughts, feelings, and actions related to one’s relationship with others and self-identity that shape how a person perceives and interprets the self (Singelis, 1994). The core of the concept is the “self-other” relationship, that is, to what extent an individual considers the self and others to be related (interdependent self-construal) or separated (independent self-construal; Cross et al., 2010). Markus and Kitayama (1991) proposed that Western culture encourages an independent self-construal that conceptualizes the self as an autonomous and bounded entity, emphasizing the independence and uniqueness of the self. In contrast, East Asian culture promotes an interdependent self-construal that conceptualizes the self as interconnected and overlapping with close others, emphasizing harmony with these close others. Studies have confirmed that individuals with different kinds of self-construals use information from different sources when making global self-evaluations. Specifically, independent self-construal uses relatively stable internal information, and interdependent self-construal uses relatively variable external information (Suh et al., 2008), which may generate distinct effects on the stability of self-evaluation (Kanagawa et al., 2001) and further influence the fear of negative evaluation and associated SA. In addition, the importance of self-construal in emotional regulation has been recognized in past decades. Studies have proven that compared to individuals with high levels of independent self-construal, those with high levels of interdependent self-construal are more likely to seek and receive social support from others, especially in collectivist cultures (Ringeisen and Buchwald, 2008; Heintzelman and Bacon, 2015). A study of the self-construal of Vietnamese–American adolescents shows that interdependent self-construal as a protective factor can help them have more family cohesion, less adverse neighborhoods, and a stronger sense of community (Lam, 2006). Ogihara and Uchida (2014) show that independent self-construal adversely predicts the number of close friends Japanese people have. Based on these findings, we speculate that different kinds of self-construals lead to varying moderated effects on the relationship between the fear of negative evaluation and SA in Chinese male individuals with drug use disorders.

Specifically, independent self-construal results in a self-definition that is “bounded, unitary, stable,” and separate from social context (Singelis, 1994). Individuals with high independent self-construal tend to think of themselves as independent of the environment to which they belong. They validate their unique internal attributes and goals and are prone to self-identification (Cross et al., 2010). They are not easily affected by the external environment and mostly analyze problems by means of decontextualization. Therefore, Chinese males with drug use disorders who exhibit a high level of independent self-construal may solidify their fear of negative evaluation, leading to the development of more severe SA. Norasakkunkit et al. (2011) confirmed the relationship between independent self-construal and SA, revealing that independent self-construal was associated with the self-focused component of SA. Individuals with a high level of independent self-construal tend to direct their attention primarily inward as a means of self-monitoring. This inward focus may amplify negative self-attributes and intensify the fear of negative evaluation, leading to excessive worry about potential embarrassment or failure to make a positive impression in public settings, thereby giving rise to SA. In terms of perceived social support, although being high in independent self-construal does not mean being disconnected from society in collectivist cultures, their loose social connections may make them lack effective social resources to act as a buffer against psychosocial problems (Barry, 2000). Studies have confirmed that independent self-construal predicts less perceived social support and more serious psychosocial problems in the cultural background of collectivism (Taniguchi and Kaufman, 2019). Therefore, we propose the following hypothesis:

*Hypothesis 2*: Independent self-construal may positively moderate the relationship between fear of negative evaluation and SA among Chinese male individuals with drug use disorders.

Interdependent self-construal is a representative personality trait in collectivist cultures; it results in self-definitions that are “flexible, variable” (Singelis, 1994), and connected to, or in harmony with, social contexts. Individuals with high interdependent self-construal tend to see themselves as intricately connected and integrated with others in their social groups. They pay more attention to social relations and others and tend to adjust to the environment to which they belong (Colzato et al., 2012). Thus, their attitude toward the environment may render them susceptible to life events and the influence of significant others. Studies have shown that the early views of the self and others are not immutable (Ammaniti et al., 2000) and that significant life events and the presence of important others are likely to contribute to the improvement of individuals’ internal working models and self-cognitions. Therefore, interdependent self-construal may help male individuals with drug use disorders obtain opportunities to ameliorate their fear of negative evaluation, thereby alleviating SA. Additionally, studies have shown that interdependent self-construal allows individuals to acquire social resources and enhance their ability to regulate psychosocial problems (Ren et al., 2013). Research on trauma-exposed American college students showed that interdependent self-construal promotes the emergence of posttraumatic growth outcomes through social support seeking (Yeung and Chow, 2019). Therefore, we propose the following hypothesis:

*Hypothesis 3*: Interdependent self-construal may negatively moderate the relationship between fear of negative evaluation and SA in Chinese male individuals with drug use disorders.

**Figure 1:**
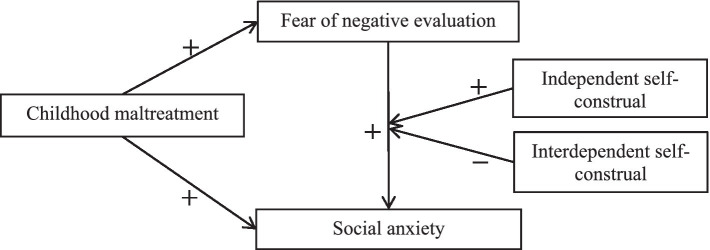
Hypothetical model.

In summary, from a sociocultural perspective, based on attachment theory and the cognitive model of SA, this study constructed a moderated mediation model, aiming to further explore the mechanism of SA among Chinese male individuals with drug use disorders. Specifically, it investigated the following: first, whether fear of negative evaluation plays a mediating role between CM and SA in Chinese male drug addicts, and second, whether the different types of self-construal may play different moderating roles in the second half path of the mediating process and whether independent self-construal positively moderates the relationship between fear of negative evaluation and SA, while interdependent self-construal does the opposite (Figure 1).

## 2 Methods

### 2.1 Participants and procedure

Individuals with drug use disorders were divided into teams (approximately 100 people per team) for daily management. The division of teams was randomized, and the present study employed cluster sampling, with teams as the units. The study included 700 male individuals with drug use disorders from two drug rehabilitation institutes in the Southwest China region. Among them, 82 participants were excluded from the analysis either because they selected the same option for more than 50% of the total items or due to missing data exceeding 10% of the total items. As a result, the final sample consisted of 618 Chinese male individuals with drug use disorders. The inclusion criteria for the study followed the definition of the Diagnostic and Statistical Manual of Mental Disorders (DSM-V) of “substance use disorder” (American Psychiatric Association, 2013). Among the participants, 546 (88.35%) were of Han ethnicity, and 72 (11.65%) belonged to minority groups. Regarding housing, 489 (79.13%) owned their houses, 94 (15.21%) rented houses, and 35 (5.66%) had changed their rented accommodations more than twice within a 6-month period. A total of 167 (27.02%) exclusively used opioids, 323 (52.27%) exclusively used methamphetamine, 67 (10.8%) engaged in opioid-dominated mixed drug use, and 61 (9.91%) engaged in methamphetamine-dominated mixed drug use. The participants’ ages ranged from 18 to 61 years, with an average age of 34.13 (SD = 8.76), and the average age of first drug use was 24.27 (SD = 7.14). The demographic characteristics of the total sample are provided in Table 1.

**Table 1:** Distribution table of demographic variables.

Variables	Classification	Quantity	Proportion (%)
Ethnic group	Han ethnicity	546	88.35
	Minorities	72	11.65
Drug type	Opioids-type	167	27.02
	Amphetamines-type	323	52.27
	Opioids-dominated mixed-type	67	10.8
	Methamphetamine-dominated mixed-type	61	9.91
Living conditions	Have their own houses	489	79.13
	Rented houses	94	15.21
	Rented houses more than twice within half a year	35	5.66

The study was approved by the Research Ethics Committee of the Sichuan Psychology Association. Before starting the test, we provided each participant with an informed consent form and obtained their consent. The participants completed the questionnaires in groups while seated in a classroom at each drug rehabilitation institute. The researchers, who were doctoral students majoring in psychology, used a standardized set of instructions to explain the requirements for completing the measures to all the participants and confirmed their understanding of the instructions while emphasizing the importance of the authenticity and completeness of their answers. The participants were not required to provide any information revealing their identity. They were reassured that their responses would be kept confidential and would have nothing to do with treatment effectiveness.

### 2.2 Materials

#### 2.2.1 Childhood maltreatment

The Childhood Trauma Questionnaire short form (CTQ-SF) was developed by Bernstein et al. (2003) and was later translated into Chinese by Zhao et al. (2005). Existing research has confirmed that the CTQ-SF demonstrates good reliability and validity when used among various population groups in China (Chen et al., 2019; He et al., 2019). The scale is a retrospective self-report questionnaire consisting of 25 clinical items that measure the extent to which respondents have experienced five categories of CM, including physical, sexual, and emotional abuse, as well as physical and emotional neglect. Each of the 5 categories of maltreatment is measured using 5 items. The participants responded on a 5-point-type scale (1 = never true to 5 = very often true) based on the frequency of each event having occurred. A quantitative score for each maltreatment category is computed and based on a validated cutoff score, and the severity of each category of maltreatment is quantified as “none or minimal,” “low to moderate,” “moderate to severe,” or “severe to extreme” (Bernstein and Fink, 1998). In this study, the cutoff scores for “low to moderate” exposure were used to examine the prevalence rates of various types of CM in Chinese male individuals with drug use disorders. The CTQ cutoff scores were as follows: physical abuse ≥ 8, sexual abuse ≥ 6, emotional abuse ≥ 9, physical neglect ≥ 8, and emotional neglect ≥ 10. The reported sensitivity and specificity for these cutoff scores reached 89% and 97%, respectively (Tietjen et al., 2010). In this study, McDonald’s ω of this scale was calculated to be 0.777.

#### 2.2.2 Fear of negative evaluation

The Fear of Negative Evaluation scale (brief version; BFNES) was developed by Leary (1983), was translated into Chinese by Chen (2002), and has demonstrated good reliability and validity when used to measure Chinese populations (Wei et al., 2015; Lee et al., 2022). The scale consists of a total of 12 items, including 8 forward-scored and 4 reverse-scored items. All the items are rated on a scale from 1 (strongly disagree) to 5 (strongly agree). A higher score indicates a greater fear experienced by the individual when threatened to be negatively evaluated by others. BFNES is widely used as a measurement tool in the field of SA. In this study, McDonald’s ω of this scale was calculated to be 0.772.

#### 2.2.3 Social anxiety

The Social Anxiety Subscale of the Self-Consciousness Scale was developed by Scheier and Carver (1985) and translated into Chinese by Wang et al. (1999), is widely used in China, and has been confirmed to possess good reliability and validity (Xu et al., 2017; You et al., 2019). The scale consists of a total of 6 items, all of which are rated on a scale from the weakest (totally not compliant = 0) to the strongest (fully compliant = 4). The situations described in the scale include unfamiliar situations, being stared at, embarrassing events, talking to strangers, public speaking, and large groups of people. The lowest score of the scale is 0, the highest score is 24, and higher scores imply more severe SA. In this study, McDonald’s ω of this scale was calculated to be 0.811.

#### 2.2.4 Self-construals

The self-construal scale (SCS) was developed by Singelis (1994) and then translated into Chinese by Wang et al. (2008), and it demonstrates good reliability and validity when used to measure Chinese populations (Wang and Wang, 2016; Li et al., 2022). It is a 24-item scale used to measure independent self-construal (12 items) and interdependent self-construal (12 items). The participants respond on a 7-point Likert scale, ranging from the weakest (strongly disagree = 1) to the strongest (strongly agree = 7). Higher scores on each dimension indicate the individual’s corresponding self-construal tendency to be more pronounced. In this study, the McDonald’s ω for the interdependent self- and independent self-dimensions was calculated to be 0.853 and 0.906, respectively.

### 2.3 Data analyses

Statistical analyses were conducted using SPSS 24.0 and PROCESS 3.0. First, the prevalence of CM was tested. Second, descriptive statistics (means and standard deviations) and correlation analyses of the study variables were computed. Third, Harman’s single-factor test was used to assess common method bias. Fourth, Hayes’s (2013) procedure was used to test the mediation model and the moderated mediation model. In the moderated mediation analysis, all variables were standardized. In addition, previous research found that the SA of individuals with drug use disorders was associated with age (Caballo et al., 2008), age at first drug use (Han et al., 2010), and drug type. We used the above variables as control variables in this study. Among them, age and age at first drug use were continuous variables. A study on SA among individuals aged 16 and above from 18 different countries, involving a total of 16,940 participants, revealed distinct patterns of significant correlations between age and various dimensions of SA (Caballo et al., 2008). Furthermore, the age at first drug use indirectly reflects the duration and severity of an individual’s drug addiction, exhibiting a significant negative correlation with the health status of individuals with drug use disorders (Han et al., 2010). Moreover, drug type was a categorical variable. In this study, drug types were categorized into four categories: opioid-type, methamphetamine-type, and mixed-type (mixed-type was further divided into opioid-dominated mixed-type and methamphetamine-dominated mixed-type). Opioids and methamphetamine are groups of illegal materials that are different from each other in terms of chemical structure, physical and psychological effects, and potential risk for individuals with drug use disorders (Parvaresh et al., 2015; Cumming et al., 2023). Methamphetamine is a highly addictive central nervous system stimulant (Haight et al., 2005), and opioids cause great damage to the central nervous system, which is manifested as excitation at first and then, mainly, inhibition (Watanabe et al., 2002). Studies have confirmed that mixed use has more serious consequences than single use (Cumming et al., 2023).

## 3 Results

### 3.1 Prevalence of childhood maltreatment

In the present study, among 618 male individuals with drug addiction, 586 (94.82%) had experienced emotional neglect, 594 (96.12%) had experienced physical neglect, 137 (22.17%) had experienced emotional abuse, 124 (20.06%) had experienced physical abuse, and 280 (45.31%) had experienced sexual abuse.

### 3.2 Common method bias

The data in this study were obtained through the self-reports of the participants. To avoid common method bias, appropriate controls, such as anonymous responding and reverse-scoring some of the items, were utilized. We also used the Harman single-factor test to conduct a common method bias test. The result of the unrotated exploratory factor analysis showed that the first factor can explain only 17.78% of the variance, far less than the critical value of 40%. This indicates that no serious common method bias was present in the data.

### 3.3 Preliminary analyses

The descriptive statistical results and correlation matrix of the variables in the current study are shown in Table 2. CM was weakly positively correlated with fear of negative evaluation and SA. Fear of negative evaluation was weakly positively correlated with SA. CM and fear of negative evaluation were weakly positively correlated with independent and interdependent self-construals. In general, the results of the correlation analysis indicated weak correlations among all variables in the study, except for the strong relationships between the SCS subscales.

**Table 2:** Means, standard deviations, and correlations of study variables (*N* = 618).

Variables	M	SD	4	5	6	7
1. Drug type	*-*	*-*				
2. Age	34.13	8.76				
3. Age of first drug use	24.29	7.14				
4. CM	50.89	6.66	1			
5. FNE	24.07	5.86	0.13^***^	1		
6. SA	6.17	2.94	0.12^**^	0.17^***^	1	
7. INDESC	4.73	1.00	0.10^**^	0.24^***^	0.02	1
8. INTDESC	4.64	0.94	0.16^***^	0.21^***^	0.02	0.75^***^

M, mean; SD, standard deviation; CM, childhood maltreatment; FNE, fear of negative evaluation; INDESC, Independent self-construal; INTDESC, Interdependent self-construal. Drug type was dummy coded.^*^p < 0.05, ^**^p < 0.01, ^***^p < 0.001.

### 3.4 Testing the mediation model

When fear of negative evaluation was the mediating variable, we used model 4 in the PROCESS Macro to test the mediation model. After controlling for age, age at first drug use, and drug type, the relationship between CM and SA revealed that CM significantly and positively predicted SA (β = 0.12, *p* < 0.01). CM significantly and positively predicted fear of negative evaluation (β = 0.12, *p* < 0.01), and the latter also significantly and positively predicted SA (β = 0.15, *p* < 0.001). After including the mediator, CM still significantly and positively predicted SA (β = 0.10, *p* < 0.01). The results of the bootstrapping analysis revealed that fear of negative evaluation partially mediated the relationship between CM and SA, and the indirect effect was 0.02, boot SE = 0.01, 95% CI = [0.005, 0.037], accounting for 16.67% of the total effect. Hypothesis 1 is verified.

### 3.5 Testing the moderated mediation model

**Figure 2:**
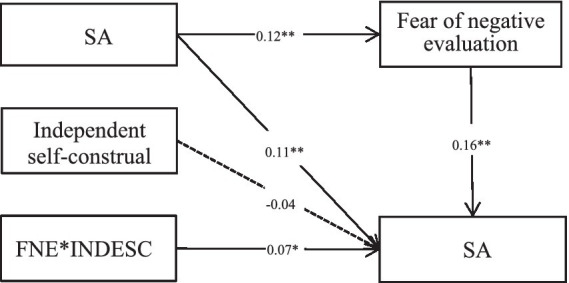
The moderated mediation effect of childhood maltreatment on social anxiety (moderator: independent self-construal). ^*^p < 0.05, ^**^p < 0.01, ^***^p < 0.001. FNE, fear of negative evaluation; INDESC, independent self-construal.

After controlling for age, age at first drug use, and drug type, we examined the moderated mediating model by using Model 14 of PROCESS Macro 3.0 for SPSS. As shown in Table 3 and Figure 2, CM significantly and positively predicted SA (β = 0.11, *p* < 0.01). In addition, fear of negative evaluation significantly and positively predicted SA (β = 0.16, *p* < 0.001), and independent self-construal had no significant effect on SA (β = −0.04, *p* > 0.05). However, the interaction between fear of negative evaluation and independent self-construal had a significant effect on SA (β = 0.07, *p* = 0.045). Hypothesis 2 is verified.

**Table 3:** The results of moderated mediation analyses (moderator: INDESC, *N* = 618).

Predictors	Mediator: FNE			Dependent variable: SA		
	β	SE	t	β	SE	t
Drug type	0.09	0.05	1.89^†^	0.07	0.05	1.56
Age	0.01	0.06	2.21^*^	−0.01	0.01	−1.14
Age of first drug use	−0.01	0.01	−1.89^†^	0.01	0.01	1.60
CM	0.12	0.04	3.03^**^	0.11	0.04	2.73^**^
FNE				0.16	0.04	3.86^***^
INDESC				−0.04	0.04	−0.87
FNE*INDESC				0.07	0.03	2.01^*^
R^2^	0.03			0.05		
F	4.87^***^			4.77^***^		

**The levels of moderator: INDESC**	**Indirect effects**	**95%LLCI**	**95%ULCI**
Low level (M − 1SD)	0.01	−0.003	0.030
High level (M + 1SD)	0.03	0.008	0.052

CM, childhood maltreatment; FNE, fear of negative evaluation; INDESC, independent self-construal; LL, lower limit; UL, upper limit; CI, confidence interval. Drug type was dummy coded. The bootstrap sample size was 5,000.^*^p < 0.05, ^**^p < 0.01, ^***^p < 0.001.

**Figure 3:**
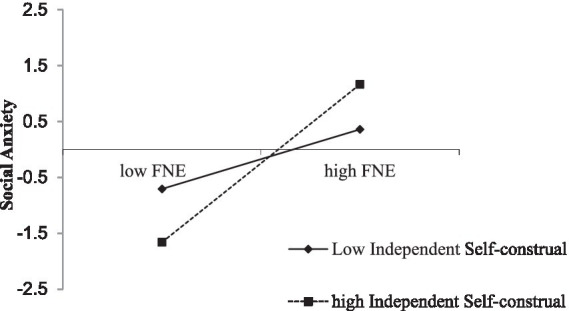
The moderating role of independent self-construal. FNE, fear of negative evaluation.

To visually show the moderating effect of independent self-construal on the relationship between fear of negative evaluation and SA, a simple slope test was conducted (Figure 3). According to the mean ± 1 standard deviation, independent self-construal was divided into high (M + 1SD) and low (M-1SD) groups, from low independent self-construal (βsimple = 0.09, SE = 0.06, 95% CI = [−0.019, 0.198]) to high independent self-construal (βsimple = 0.23, SE = 0.05, 95% CI = [0.125, 0.332]), and the effect of fear of negative evaluation on SA changed from insignificant to significant.

When the mediating process was moderated, it was necessary to check whether the indirect effect changed with the moderating variable (Table 3). The indirect effect was stronger at high (M + 1 SD) independent self-construal (β = 0.03, Boot SE = 0.012, 95% CI = [0.008, 0.052]) than at low (M − 1SD) independent self-construal (β = 0.01, Boot SE = 0.008, 95% CI = [−0.003, 0.030]). For low independent self-construal, the indirect effect value accounted for 8.3% of the total effect; for high independent self-construal, it accounted for 25% of the total effect. These results indicate that independent self-construal positively moderated the indirect effect of CM on SA in male individuals with drug use disorders via fear of negative evaluation.

We used the same method to construct a moderated mediation model to test the moderated effect of interdependent self-construal on the second half path of the mediation model. The results show that interdependent self-construal (β = −0.04, *p* = 0.31) and the interaction between fear of negative evaluation and interdependent self-construal (β = 0.05, *p* = 0.14) had no significant effect on SA. That is, interdependent self-construal had no moderating effect on the link between fear of negative evaluation and SA, and Hypothesis 3 was unconfirmed.

## 4 Discussion

This study confirms that CM has not only a direct positive relationship with SA but also an indirect relationship with SA through the mediating factor of fear of negative evaluation among Chinese male individuals with drug use disorders. In addition, independent self-construal positively moderates the indirect association between CM and SA among Chinese male individuals with drug use disorders via the fear of negative evaluation. Specifically, the indirect association between CM and SA is stronger among individuals with high independent self-construal than among those with low independent self-construal. The results of this study help to explain the mechanism of SA in Chinese male individuals with drug use disorders and demonstrate a certain theoretical and practical significance in alleviating their SA.

The mediation analysis indicates that CM is not only directly associated with SA in Chinese male individuals with drug use disorders but can also be indirectly linked to SA through the mediation of fear of negative evaluation. The integration of CM as complex trauma with the causal mechanisms of internal working models and the cognitive model of SA can provide an explanatory framework for these findings. Specifically, CM, as a form of complex trauma, provides ample conditions for the development of negative self- and others’ cognition, given its negative impact on self-concept, emotional regulation, and interpersonal relationships (Courtois, 2004; Maercker, 2021). These serve as a significant foundation for the emergence of SA. Shahar et al. (2015) established a sequential mediation model in a non-clinical adult sample, demonstrating that childhood emotional abuse predicted shame proneness, which, in turn, predicted self-criticism, and self-criticism subsequently predicted symptoms of SA. These findings offer preliminary evidence that supports the role of negative emotions and negative self-concept in the development and maintenance of SA among individuals with a history of CM. Wang et al. (2022) confirmed that emotional dysregulation can explain the relationship between harsh parenting by parents and adolescent SA. Based on the perspective of attachment theory, early caregiving experiences create internalized representations of interpersonal relationships, and thus, many interpersonal issues are closely associated with CM. Fitzgerald (2022) confirmed that CM has a significant indirect impact on SA symptoms through relationship quality. Moreover, CM, as a negative form of early parent–child interaction, can result in insecure attachment, prompting individuals to process information in a schema-driven manner consistent with their negative attachment experiences (Dykas and Cassidy, 2011). This processing pattern contributes to the formation of negative internal working models, which also provide the conditions for the development of fear of negative evaluation. Fear of negative evaluation is the core cognitive component of SA, which makes individuals prioritize the allocation of attention resources to detect social threats in the environment, monitor the environment to find evidence of negative evaluation, and monitor themselves to find defects that may cause others’ negative evaluations (MacLeod and Mathews, 1991). In summary, negative self- and others’ cognition and internal working models resulting from CM lead to the establishment of a relationship between CM and the development of fear of negative evaluation and SA.

Second, the study results indicate that independent self-construal moderates the association between fear of negative evaluation and SA among Chinese male individuals with drug use disorders. We found that as the level of independent self-construal increased, the positive association between fear of negative evaluation and SA also increased. This result is consistent with our research hypothesis. However, previous research has confirmed a negative correlation between independent self-construal and SA. The contradictions in the research results mainly stem from differences in cultural backgrounds. In individualistic cultures, a negative relationship between independent self-construal and SA is confirmed (Park et al., 2011; Krieg and Xu, 2018), while in collectivistic cultures, a positive correlation is observed (Xie et al., 2008). Self-construal is shaped within the cultural context, so an incongruence between these two aspects often signifies a form of maladaptation. When an individual’s self-construal is out of sync with their cultural milieu, they may encounter a series of adaptation challenges. These challenges can encompass diminished social acceptance, increased social pressure, and the inability to meet the expectations set by their culture’s norms, leading to negative self-view, emotional distress, and psychological discomfort (Sakman and Sümer, 2022). Consequently, the conflict between self-construal and the cultural environment is commonly regarded as an indicator of adaptation issues. Norasakkunkit and Uchida (2011) confirmed that independent self-construal serves as an indicator of social adaptation difficulties within a collectivist cultural context, and a high level of independent self-construal often reflects interpersonal adaptation issues among individuals in collectivist cultures. A study on cross-cultural differences in social emotions showed that in cultures with a stronger collectivistic orientation, independent self-construal was negatively associated with positive affect (Nezlek et al., 2008). Additionally, the strong self-identification tendency and decontextualized thinking patterns associated with a high level of independent self-construal result in individuals having a stable self-view (Singelis, 1994). Consequently, Chinese male individuals with drug use disorders characterized by a high level of independent self-construal may be inclined to collect more evidence along with their pre-existing negative self-definition to maintain or exacerbate their fear of negative evaluation and associated SA. At the same time, studies have proven that the loose social connection of independent self-construal makes individuals lack enough social support to resist the influence of negative social emotions in the cultural background of collectivism (Taniguchi and Kaufman, 2019). Based on the above analysis, with the increase in independent self-construal, the fear of negative evaluation is more stable or even aggravated, contributing to the development and maintenance of SA.

Markus and Kitayama (1991) proposed that collectivist cultures promote an interdependent self-construal that conceptualizes the self as interconnected and overlapping with close others, emphasizing harmony with these close others. Individuals with high interdependent self-construal are characterized by their connectedness and coordination with the social environment, demonstrating a “flexible” and “adaptable” self-view (Singelis, 1994; Colzato et al., 2012). Research has demonstrated that a close social connection with high interdependent self-construal can help individuals acquire social resources more easily and enhance their ability to regulate social emotions, especially in the context of collectivist cultures (Ren et al., 2019). Ren et al. (2013) demonstrated through experimental research that an interdependent self-construal enables people from collectivistic cultures to recover from the negative effects of interpersonal rejection. At the same time, the compatibility between an individual’s self-construal and their cultural environment, particularly the alignment of the primary components of self-construal with the cultural context, typically facilitates better adaptation and reduces the likelihood of interpersonal difficulties (Sakman and Sümer, 2022). Based on the traits of interdependent self-construal, we speculate that it may negatively moderate the relationship between the fear of negative evaluation and SA of Chinese male individuals with drug use disorders. However, the aforementioned hypothesis has not been corroborated in this study. Through further analysis, we found that the self-construal characteristics of the participants in this study were incongruent with those typically observed in a collectivist cultural background. Specifically, their scores on independent self-construal were significantly higher than their scores on interdependent self-construal (t = −3.323, *p* = 0.001). This indicates that the participants in this study exhibited weaker traits related to interdependent self-construal. This incongruity may explain why the moderating effect of interdependent self-construal on the latter part of the mediating model pathway was not significant.

Individuals with a collectivist cultural background are expected to exhibit a higher degree of interdependent self-construal. The self-construal characteristics observed in Chinese male drug use disorders in this study are incongruent with the collectivist cultural background and may be indicative of sociocultural deviance. A study of Hikikomori in Japan confirms that self-construal is an index of sociocultural deviance, and groups with sociocultural deviance show high independent self-construal, which is particularly prominent in cultures that emphasize collectivism (Norasakkunkit and Uchida, 2011). Sociocultural deviance theory suggests that the tendency to deviate from mainstream sociocultural attitudes and values is a risk factor for interpersonal adaptation problems (Norasakkunkit and Uchida, 2014). Studies have proven that sociocultural deviance is closely associated with a negative self-view and deviant behaviors (Wells, 1978; Torkaman et al., 2020; Alison et al., 2021). It may reduce the extent to which individuals with drug use disorders see themselves as members of society and increase the probability of deviant behaviors. These two aspects can influence each other, gradually leading them to adopt deviant values, thus inducing deviant self-identification and further reinforcing the degree of sociocultural deviance. In fact, a negative view of the self and sociocultural deviance are mutually causal, creating a vicious circle that leads to the alienation of individuals with drug addiction from society. The attitudes of society toward Chinese male individuals with drug use disorders may serve as a breakthrough point for breaking this vicious cycle. Specifically, understanding and destigmatizing assessments from the general public have the potential to ameliorate the extent of sociocultural deviance among Chinese male drug addicts, enhance their negative self-evaluations, reduce their levels of SA, and decrease the probability of engaging in deviant behaviors. However, there has been a persistent tendency among the Chinese general public to hold relatively negative perceptions of drug use disorders, with biases and stigma being widespread (Luo et al., 2014), which can potentially pose a barrier for individuals with substance addiction, confining them to a subculture diverging from mainstream culture and impeding their social adaptation. Therefore, it may be evident that the social stigma and prejudice faced by Chinese individuals with drug use disorders are significant influencing factors that need to be considered when addressing the social adaptation of this population. Based on sociocultural deviance theory, the high independent self-construal of Chinese male individuals with drug use disorders in this study may indicate their sociocultural deviance and associated negative view of the self and others. This belief in negative evaluations from social situations strengthens the fear of negative evaluation, which further reinforces their SA.

## 5 Limitations and future research directions

Some limitations of this study merit exploration in future research. First, this study exclusively focused on male individuals with drug use disorders in China. While the findings offer insights into understanding SA within this group, the limitation of the study’s sample being exclusively male restricts the generalizability of the results. Therefore, in future research, we will first consider expanding the participant pool to include female individuals and possibly other racial or cultural groups. Second, the research subjects of this study were all individuals with drug use disorders in the isolation detoxification stage, and their interpersonal situations were distorted, which might have influenced the test results to some extent. Future research should include individuals with drug use disorders in community detoxification programs, which would increase the ecological validity of the study. Third, although the variables we selected for inclusion are grounded in theory, our research design remained cross-sectional. Fairchild and McDaniel (2017) concluded in their study that examining mediation in cross-sectional data implicitly undermines an assumption of the statistical mediation model: the presumption that the temporal ordering of variables in the causal chain of mediation is correct. Thus, any estimations of a mediation effect in such data are fundamentally correlated in nature. Hence, it is advisable for readers to interpret the study results regarding variable relationships with caution. Moreover, conducting longitudinal studies or experimental research with the correct temporal ordering of variables is the direction for our future research efforts to further explore the relationships between variables. Fourth, in retrospective self-report measurements, there is a possibility of recall bias that can affect the measurement results, for instance, in the assessment of CM. Previous research has demonstrated that retrospective reports of CM can impact the validity of findings regarding the association between childhood adversity and mental illness (Gayer-Anderson et al., 2020). The source of recall bias lies in autobiographical memory (Kuyken and Brewin, 1995), which is not an objective account of events but is subject to reconstructive and selective processes related to the self, leading individuals to recall their past experiences influenced by subsequent life events during development. Thus, this research suggests that, in future studies on CM among individuals with drug use disorders, structured interviews should be incorporated as a supplement to enhance the quality of measuring CM in this population. Fifth, based on the characteristics of fear of negative evaluation, combined with the issue of small effect sizes in the research, we speculate that the focus of this study on explicit fear of negative evaluations may not capture the complete extent of fear of negative evaluations in this population. Exploring the implicit fear of negative evaluations in individuals with drug addiction is a research topic worthy of attention. Sixth, this study extensively cites concepts such as attachment theory and social support to justify the relationships between these variables. However, the utilization of these theories and variables in this study is still at the theoretical confirmation stage, and these variables have not been measured. Given the substantial value of these theories and concepts to research in this field, we intend to measure and analyze these variables in future studies, empirically substantiating their support for the hypotheses presented in the current research. Seventh, the current study has an underlying research question, namely, whether the high prevalence of CM among individuals with drug use disorders may be attributed to the impact of using drugs as a means to cope with post-traumatic symptoms resulting from CM experiences. Although this specific issue was not examined in this article, it will be a research direction to enhance our understanding of the relationship between CM and drug use. Eighth, this study extrapolates potential sociocultural deviations among Chinese male individuals with drug use disorders based on the characteristics of their self-construal. It suggests that these deviations need to be further validated in future research, and the factors influencing the occurrence of sociocultural deviations in Chinese individuals with drug use disorders need to be explored. This holds significant and far-reaching implications for a deeper understanding of social adaptation issues within the drug addiction population and the proposal of effective strategies.

## 6 Implications

Although the present study has certain limitations, the relevant results nonetheless have significant implications. First, our findings prove that the past life experiences of individuals with drug use disorders influence their social adjustment. Therefore, a relatively personalized and targeted social adjustment intervention and guidance program based on the adverse life experiences of individuals with drug use disorders is the direction we are working toward. Self-construal is the innovation considered in this work. Previous studies have proven that self-construal can be primed and shaped. Therefore, in the later stage, based on relevant research, we can try to link treatment to the self-construal traits of individuals with drug use disorders to formulate a plan to shape their self-construals. At the same time, social discrimination and stigma may act with traumatic childhood experiences against drug addicts, further enhancing the sociocultural deviation of the group and thereby inducing deviant self-identification. Therefore, public awareness campaigns to prevent discrimination and stigma toward drug addicts are necessary to alleviate their sociocultural deviation, which may help improve their social adjustment.

## 7 Conclusion

In conclusion, the findings from this study deepen the understanding of the relationship between the CM and SA of Chinese male individuals with drug use disorders. Specifically, CM has not only a direct positive relationship with the SA of Chinese male individuals with drug use disorders but also an indirect relationship with SA through the mediating factor of fear of negative evaluation. Additionally, independent self-construal moderates the indirect predictive association between CM and SA via the fear of negative evaluation. Moreover, the gap in the scores of the two kinds of self-construal among Chinese male individuals with drug use disorders suggests that the social adjustment of this group may be affected by sociocultural deviance. These findings may help researchers and practitioners by providing insight into future social adaptation interventions for this discussed population, which need to be individualized and implemented for those who have experienced CM, and self-construal provides a path for that work.

## Data availability statement

The original contributions presented in the study are included in the article/supplementary material, further inquiries can be directed to the corresponding author.

## Ethics statement

The studies involving humans were approved by the Research Ethics Committee of the Sichuan Psychology Association. The studies were conducted in accordance with the local legislation and institutional requirements. The participants provided their written informed consent to participate in this study.

## Author contributions

YL: conceptualization, methodology, data collection and analysis, writing–original draft, and writing–review. HZ: data collection. HM: revisions. JZ: revisions. CG: revisions and supervision. All authors contributed to the article and approved the submitted version.

## References

[ref1] Abbasi M. A. Saeidi M. Khademi G. Hoseini B. L. Moghadam Z. E. (2015). Child maltreatment in the worldwide: a review article. Int. J. Pediatr. 3, 353–365. doi: 10.22038/ijp.2015.3753

[ref2] Aff T. O. Henriksen C. A. Asmundson G. J. Sareen J. (2012). Childhood maltreatment and substance use disorders among men and women in a nationally representative sample. Can. J. Psychiatry 57, 677–686. doi: 10.1177/070674371205701105 23149283

[ref3] Agosti V. Nunes E. Levin F. (2002). Rates of psychiatric comorbidity among US residents with lifetime cannabis dependence. Am. J. Drug Alcohol Abuse 28, 643–652. doi: 10.1081/ada-120015873, PMID: 12492261

[ref4] Alison F. W. W. Tai L. C. Caroline C. Jennifer Y. F. L. (2021). Understanding the links between self-concept, sociocultural deviance and mental health problems in pathological social withdrawal. Curr. Psychol. 42, 5290–5296. doi: 10.1007/s12144-021-01865-7

[ref5] American Psychiatric Association (2013). “Substance use disorder” in Diagnostic and statistical manual of mental disorders. 5th ed (Washington, DC: American Psychiatric Pub), 483–550.

[ref6] Ammaniti M. Van Ijzendoorn M. H. Speranza A. M. Tambelli R. (2000). Internal working models of attachment during late childhood and early adolescence: an exploration of stability and change. Attach. Hum. Dev. 2, 328–346. doi: 10.1080/14616730010001587, PMID: 11708222

[ref7] Bailey H. N. Moran G. Pederson D. R. (2007). Childhood maltreatment, complex trauma symptoms, and unresolved attachment in an at-risk sample of adolescent mothers. Attach. Hum. Dev. 9, 139–161. doi: 10.1080/14616730701349721, PMID: 17508314

[ref8] Barry D. T. (2000). East Asians in America: Relationships between ethnic identity, self-construal, mental health and acculturation patterns in east Asian immigrants. Toledo, OH: University of Toledo Press.

[ref9] Beeney J. E. Stepp S. D. Hallquist M. N. Scott L. N. Wright A. G. C. Ellison W. D.. (2015). Attachment and social cognition in borderline personality disorder: specificity in relation to antisocial and avoidant personality disorders. Personal. Disord. Theory Res. Treat. 6, 207–215. doi: 10.1037/per0000110, PMID: 25705979 PMC4509943

[ref10] Bernstein D. P. Fink L. (1998). Childhood trauma questionnaire: A retrospective self-report: Manual. New York: Psychological Corporation.

[ref11] Bernstein D. P. Stein J. A. Newcomb M. D. Walker E. Pogge D. Ahluvalia T.. (2003). Development and validation of a brief screening version of the childhood trauma questionnaire. Child Abuse Negl. 27, 169–190. doi: 10.1016/s0145-2134(02)00541-0, PMID: 12615092

[ref12] Bretherton I. Munholland K. A. (1999). “Internal working models in attachment relations: a construct revisited” in Handbook of attachment: Theory, research, and clinical applications. eds. Cassidy J. Shaver P. R. (New York: Guilford), 89–111.

[ref13] Brook C. A. Schmidt L. A. (2008). Social anxiety disorder: a review of environmental risk factors. Neuropsychiatr. Dis. Treat. 4, 123–143. doi: 10.2147/ndt.s1799 18728768 PMC2515922

[ref14] Buckner J. D. Zvolensky M. J. Ecker A. H. Jeffries E. R. (2016). Cannabis craving in response to laboratory-induced social stress among racially diverse cannabis users: the impact of social anxiety disorder. J. Psychopharmacol. 30, 363–369. doi: 10.1177/0269881116629115 26839322 PMC5147424

[ref15] Buckner J. D. Zvolensky M. J. Schmidt N. B. (2012). Cannabis-related impairment and social anxiety: the roles of gender and cannabis use motives. Addict. Behav. 37, 1294–1297. doi: 10.1016/j.addbeh.2012.06.013 22766487

[ref16] Caballo V. E. Salazar I. C. Irurtia M. J. Arias B. Hofmann S. G. CISO-A Research Team (2008). Social anxiety in 18 nations: sex and age differences. Behav Psychol. 16, 163–187. doi: 10.1016/j.paid.2014.02.013

[ref17] Cândea D. M. Szentagotai-Tătar A. (2018). Shame-proneness, guilt-proneness and anxiety symptoms: a meta-analysis. J. Anxiety Disord. 58, 78–106. doi: 10.1016/j.janxdis.2018.07.005, PMID: 30075356

[ref18] Chen Z. Y. (2002). Fear of negative evaluation and test anxiety in middle school students. J Chin Mental Health 16, 855–857.

[ref19] Chen Y. Zhang J. Sun Y. (2019). The relationship between childhood abuse and depression in a sample of Chinese people who use methamphetamine. Int. J. Clin. Health Psychol. 19, 181–188. doi: 10.1016/j.ijchp.2019.06.003, PMID: 31516496 PMC6732768

[ref20] Chirkov V. (2020). The sociocultural movement in psychology, the role of theories in sociocultural inquiries, and the theory of sociocultural models. Asian J. Soc. Psychol. 23, 119–134. doi: 10.1111/ajsp.12409

[ref21] Cicchetti D. Valentino K. (2006). “An ecological-transactional perspective on child maltreatment: failure of the average expectable environment and its inflfluence on child development” in Developmental psychopathology Vol. 3: Risk, disorder, and adaptation. eds. Cicchetti D. Cohen D. J.. 2nd ed (New Jersey: Wiley & Sons), 129–201.

[ref22] Cloitre M. Hyland P. Bisson J. I. Brewin C. R. Roberts N. P. Karatzias T.. (2019). ICD-11 posttraumatic stress disorder and complex posttraumatic stress disorder in the United States: a population-based study. J. Trauma. Stress 32, 833–842. doi: 10.3402/ejpt.v4i0.20706, PMID: 31800131

[ref23] Collins K. A. Westra H. A. Dozois D. J. Stewart S. H. (2005). The validity of the brief version of the fear of negative evaluation scale. J. Anxiety Disord. 19, 345–359. doi: 10.1016/j.janxdis.2004.02.003, PMID: 15686861

[ref24] Colzato L. S. de Bruijn E. R. Hommel B. (2012). Up to “me” or up to “us”? The impact of self-construal priming on cognitive self-other integration. Front. Psychol. 3:341. doi: 10.3389/fpsyg.2012.00341, PMID: 23049518 PMC3442283

[ref25] Courtois C. A. (2004). Complex trauma, complex reactions: assessment and treatment. Psychotherapy 41, 412–425. doi: 10.1037/0033-3204.41.4.412

[ref26] Cross S. E. Hardin E. E. Gercek-Swing B. (2010). The what, how, why, and where of self-construal. Pers. Soc. Psychol. Rev. 15, 142–179. doi: 10.1177/1088868310373752, PMID: 20716643

[ref27] Cumming C. Kinner S. A. McKetin R. Li I. Preen D. B. (2023). The health needs of people leaving prison with a history of methamphetamine and/or opioid use. Drug Alcohol Rev. 42, 778–784. doi: 10.1111/dar.13636 36917515 PMC10947398

[ref28] Devi R. Anand S. Shekhar C. (2013). Abuse and neglect as predictors of self concept among below poverty line adolescents from India. Int J Psychol Counsel 5, 122–128. doi: 10.5897/IJPC2013.0213

[ref29] DiLillo D. Lewis T. Loreto-Colgan A. D. (2007). Child maltreatment history and subsequent romantic relationships: exploring a psychological route to dyadic difculties. J. Aggress. Maltreat. Trauma 15, 19–36. doi: 10.1300/J146v15n01_02

[ref30] Dykas M. J. Cassidy J. (2011). Attachment and the processing of social information across the life span: theory and evidence. Psychol. Bull. 137, 19–46. doi: 10.1037/a0021367, PMID: 21219056

[ref31] Fairchild A. J. McDaniel H. L. (2017). Best (but oft-forgotten) practices: mediation analysis. Am. J. Clin. Nutr. 105, 1259–1271. doi: 10.3945/ajcn.117.152546, PMID: 28446497 PMC5445681

[ref32] Feeny B. C. Cassidy J. Ramos-Marcuse F. (2008). The generalization of attachment representations to new social situations: predicting behavior during initial interactions with strangers. J. Pers. Soc. Psychol. 95, 1481–1498. doi: 10.1037/a0012635 19025297 PMC2593839

[ref33] Fitzgerald M. (2022). A drive for redemption: relationship quality as a mediator linking childhood maltreatment to symptoms of social anxiety and depression. J. Adult Dev. 29, 29–39. doi: 10.1007/s10804-021-09383-3

[ref34] Flynn M. Cicchetti D. Rogosch F. (2014). The prospective contribution of childhood maltreatment to low self-worth, low relationship quality, and symptomatology across adolescence: a developmental-organizational perspective. Dev. Psychol. 50, 2165–2175. doi: 10.1037/a0037162, PMID: 25046123 PMC4167675

[ref35] Ford J. D. Courtois C. A. (2021). Complex PTSD and borderline personality disorder. Borderline Personal Disorder Emot Dysregul 8:16. doi: 10.1186/s40479-021-00155-9, PMID: 33958001 PMC8103648

[ref36] Formiga M. B. Galdino M. K. C. Vasconcelos S. C. Neves J. W. Lima M. D. D. C. (2021). Executive functions and emotion regulation in substance use disorder. J. Bras. Psiquiatr. 70, 236–244. doi: 10.1590/0047-2085000000331

[ref37] Garety P. A. Kuipers E. Fowler D. Freeman D. Bebbington P. E. (2001). A cognitive model of the positive symptoms of psychosis. Psychol. Med. 31, 189–195. doi: 10.1017/S0033291701003312 11232907

[ref38] Gayer-Anderson C. Reininghaus U. Paetzold I. Hubbard K. Beards S. Mondelli V.. (2020). A comparison between self-report and interviewer-rated retrospective reports of childhood abuse among individuals with first-episode psychosis and population-based controls. J. Psychiatr. Res. 123, 145–150. doi: 10.1016/j.jpsychires.2020.02.002 32065950 PMC7054833

[ref39] Grenyer B. F. S. Williams G. Swift W. Neill O. (1992). The prevalence of social-evaluative anxiety in opioid users seeking treatment. Int. J. Addict. 27, 665–673. doi: 10.3109/10826089209068758, PMID: 1319414

[ref40] Grice D. E. Brady K. T. Dustan L. R. Malcolm R. Kilpatrick D. G. (1995). Sexual and physical assault history and post-traumatic stress disorder in substance-dependent individuals. Am. J. Addict. 4, 297–305. doi: 10.3109/10550499508997446

[ref41] Griffin D. Bartholomew K. (1994). Models of the self and other: fundamental dimensions underlying measures of adult attachment. J. Pers. Soc. Psychol. 67, 430–445. doi: 10.1037/0022-3514.67.3.430

[ref42] Haight W. Jacobsen T. Black J. Kingery L. Sheridan K. Mulder C. (2005). “In these bleak days”: parent methamphetamine abuse and child welfare in the rural Midwest. Child Youth Serv. Rev. 27, 949–971. doi: 10.1016/j.childyouth.2004.12.025

[ref43] Han B. Gfroerer J. C. Colliver J. D. (2010). Associations between duration of illicit drug use and health conditions: results from the 2005–2007 National Surveys on drug use and health. Ann. Epidemiol. 20, 289–297. doi: 10.1016/j.annepidem.2010.01.003, PMID: 20171900

[ref44] Han S. Humphreys G. (2016). Self-construal: a cultural framework for brain function. Curr. Opin. Psychol. 8, 10–14. doi: 10.1016/j.copsyc.2015.09.013 29506783

[ref45] Hayes A. F. (2013). Introduction to mediation, moderation, and conditional process analysis: A regression-based approach. New York, NY: Guilford Press.

[ref46] He J. Zhong X. Gao Y. Xiong G. Yao S. (2019). Psychometric properties of the Chinese version of the childhood trauma questionnaire-Short form (CTQ-SF) among undergraduates and depressive patients. Child Abuse Negl. 91, 102–108. doi: 10.1016/j.chiabu.2019.03.009, PMID: 30856597

[ref47] Heimberg R. G. Brozovich F. A. Rapee R. M. (2010). “A cognitive-behavioral model of social anxiety disorder: update and extension” in Social anxiety: Clinical, developmental, and social perspectives. eds. Hofmann S. G. DiBartolo P. M. (New York: Elsevier).

[ref48] Heintzelman S. J. Bacon P. L. (2015). Relational self-construal moderates the effect of social support on life satisfaction. Personal. Individ. Differ. 73, 72–77. doi: 10.1016/j.paid.2014.09.021

[ref49] Herman J. (2012). CPTSD is a distinct entity: comment on Resick et al. (2012). J. Trauma. Stress 25, 256–257. doi: 10.1002/jts.21697, PMID: 22729977

[ref50] Herman J. L. (2015). Trauma and recovery: The aftermath of violence—From domestic abuse to political terror. London: Hachette UK.

[ref51] Huh H. J. Kim K. H. Lee H. K. Chae J. H. (2017). The relationship between childhood trauma and the severity of adulthood depression and anxiety symptoms in a clinical sample: the mediating role of cognitive emotion regulation strategies. J. Affect. Disord. 213, 44–50. doi: 10.1016/j.jad.2017.02.009 28189964

[ref52] Kanagawa C. Cross S. E. Markus H. R. (2001). “Who am I?” the cultural psychology of the conceptual self. Pers. Soc. Psychol. Bull. 27, 90–103. doi: 10.1177/0146167201271008

[ref53] Kessler R. C. McGonagle K. A. Zhao S. Nelson C. B. Hughes M. Eshleman S.. (1994). Lifetime and 12-month prevalence of DSM-III-R psychiatric disorders in the United States: results from the National Comorbidity Survey. Arch. Gen. Psychiatry 51, 8–19. doi: 10.1001/archpsyc.1994.03950010008002, PMID: 8279933

[ref54] Khoury L. Tang Y. L. Bradley B. Cubells J. F. Ressler K. J. (2010). Substance use, childhood traumatic experience, and post-traumatic stress disorder in an urban civilian population. Depress. Anxiety 27, 1077–1086. doi: 10.1002/da.20751, PMID: 21049532 PMC3051362

[ref55] Kong J. Moorman S. M. Martire L. M. Almeida D. M. (2019). The role of current family relationships in associations between childhood abuse and adult psychological functioning. J Gerontol Series B 74, 858–868. doi: 10.1093/geronb/gby076, PMID: 29924362 PMC6566329

[ref56] Krieg A. Xu Y. (2018). From self-construal to threat appraisal: understanding cultural differences in social anxiety between Asian Americans and European Americans. Cultur. Divers. Ethnic Minor. Psychol. 24, 477–488. doi: 10.1037/cdp0000194, PMID: 29781633

[ref57] Kuo J. R. Goldin P. R. Werner K. Heimberg R. G. Gross J. J. (2011). Childhood trauma and current psychological functioning in adults with social anxiety disorder. J. Anxiety Disord. 25, 467–473. doi: 10.1016/j.janxdis.2010.11.011, PMID: 21183310 PMC3074005

[ref58] Kuyken W. Brewin C. R. (1995). Autobiographical memory functioning in depression and reports of early abuse. J. Abnorm. Psychol. 104, 585–591. doi: 10.1037/0021-843X.104.4.585, PMID: 8530760

[ref59] Lam B. T. (2006). Self-construal and socio-emotional development among Vietnamese-American adolescents: an examination of different types of self-construal. Int. J. Behav. Dev. 30, 67–75. doi: 10.1177/0165025406062125

[ref60] Lan X. F. Zhang J. F. (2015). The gender difference of FNE: based on the comparison of implicit and explicit measurement. J Psychol Behav Res 1, 25–30.

[ref61] Leary M. R. (1983). A brief version of the fear of negative evaluation scale. Pers. Soc. Psychol. Bull. 9, 371–375. doi: 10.1177/0146167283093007

[ref62] Lee A. Chan W. Ng J. C. K. (2022). The role of fear of negative evaluation on the effects of self-control on affective states and life satisfaction: a moderated mediation analysis. Curr. Psychol. 1-14, 1–14. doi: 10.1007/s12144-022-04130-7, PMID: 36570054 PMC9762663

[ref63] Lewis C. Koyasu M. Oh S. Ogawa A. Short B. Huang Z. (2009). Culture, executive function, and social understanding. New Dir. Child Adolesc. Dev. 2009, 69–85. doi: 10.1002/cd.236 19306275

[ref64] Li J. Xue W. Zhao J. Tan L. (2022). Cognitive bias and fear of missing out (FOMO) among Chinese college students: the mediating effects of attentional control, need to belong and self-construal. Curr. Psychol. 42, 23123–23132. doi: 10.1007/s12144-022-03435-x

[ref65] Ling W. Fang L. Wang C. (2005). A Cross-cultural comparison of stress-related issues among Chinese and American college students. Chin J Psychol Health 5

[ref66] Lucero M. M. Satz S. Miceli R. Swartz H. A. Manelis A. (2022). The effects of mood disorders and childhood trauma on fear of positive and negative evaluation. Acta Psychol. (Amst) 227:103603. doi: 10.1016/j.actpsy.2022.103603, PMID: 35523082 PMC9189689

[ref67] Luo T. Wang J. Li Y. Wang X. Tan L. Deng Q.. (2014). Stigmatization of people with drug dependence in China: a community-based study in Hunan province. Drug Alcohol Depend. 134, 285–289. doi: 10.1016/j.drugalcdep.2013.10.015 24239068

[ref68] MacLeod C. Mathews A. (1991). Biased cognitive operations in anxiety: accessibility of information or assignment of processing priorities? Behav. Res. Ther. 29, 599–610. doi: 10.1016/0005-7967(91)90009-R 1759958

[ref69] Maercker A. (2021). Development of the new CPTSD diagnosis for ICD-11. Borderline Personal Disord Emot Dysregul 8, 7–4. doi: 10.1186/s40479-021-00148-8, PMID: 33641675 PMC7919312

[ref70] Markus H. R. Kitayama S. (1991). “Cultural variation in the self-concept” in The self: Interdisciplinary approaches. eds. Witkin H. A. Butterworth G. H. (New York, NY: Springer New York), 18–48.

[ref71] Morrison A. P. Frame L. Larkin W. (2003). Relationships between trauma and psychosis: a review and integration. Br. J. Clin. Psychol. 42, 331–353. doi: 10.1348/014466503322528892, PMID: 14633411

[ref72] Morrison A. S. Heimberg R. G. (2013). Social anxiety and social anxiety disorder. Annu. Rev. Clin. Psychol. 9, 249–274. doi: 10.1146/annurev-clinpsy-050212-185631 23537485

[ref73] Moscovitch D. A. (2009). What is the core fear in social phobia? A new model to facilitate individualized case conceptualization and treatment. Cogn. Behav. Pract. 16, 123–134. doi: 10.1016/j.cbpra.2008.04.002

[ref75] Nezlek J. B. Kafetsios K. Smith C. V. (2008). Emotions in everyday social encounters: correspondence between culture and self-construal. J. Cross Cult. Psychol. 39, 366–372. doi: 10.1177/0022022108318114

[ref76] Ng A. S. Abbott M. J. Hunt C. (2014). The effect of self-imagery on symptoms and processes in social anxiety: a systematic review. Clin. Psychol. Rev. 34, 620–633. doi: 10.1016/j.cpr.2014.09.003 25455626

[ref77] Norasakkunkit V. Kalick S. M. (2009). Experimentally detecting how cultural differences on social anxiety measures misrepresent cultural differences in emotional well-being. J. Happiness Stud. 10, 313–327. doi: 10.1007/s10902-007-9082-1

[ref78] Norasakkunkit V. Kitayama S. Uchida Y. (2011). Social anxiety and holistic cognition: self-focused social anxiety in the United States and other-focused social anxiety in Japan. J. Cross Cult. Psychol. 43, 742–757. doi: 10.1177/0022022111405658

[ref79] Norasakkunkit V. Uchida Y. (2011). Psychological consequences of postindustrial anomie on self and motivation among Japanese youth. J. Soc. Issues 67, 774–786. doi: 10.1111/j.1540-4560.2011.01727.x

[ref80] Norasakkunkit V. Uchida Y. (2014). To conform or to maintain self-consistency? Hikikomori risk in Japan and the deviation from seeking harmony. J. Soc. Clin. Psychol. 33, 918–935. doi: 10.1521/jscp.2014.33.10.918

[ref81] Ogihara Y. Uchida Y. (2014). Does individualism bring happiness? Negative effects of individualism on interpersonal relationships and happiness. Front. Psychol. 5:135. doi: 10.3389/fpsyg.2014.00135, PMID: 24634663 PMC3942875

[ref82] Oikawa S. Yoshida T. (2007). An identity based on being different: a focus on Biethnic individuals in Japan. Int. J. Intercult. Relat. 31, 633–653. doi: 10.1016/j.ijintrel.2007.05.001

[ref83] Park I. J. Sulaiman C. Schwartz S. J. Kim S. Y. Ham L. S. Zamboanga B. L. (2011). Self-construals and social anxiety among Asian American college students: testing emotion suppression as a mediator. Asian Am. J. Psychol. 2, 39–50. doi: 10.1037/a0023183, PMID: 33708347 PMC7946160

[ref84] Parvaresh N. Mazhari S. Nazari-Noghabi M. (2015). Frequency of psychiatric disorders in children of opioid or methamphetamine-dependent patients. Addict. Health. 7, 140–148. doi: 10.22122/AHJ.V7I3-4.326 26885350 PMC4741234

[ref85] Penner F. Gambin M. Sharp C. (2019). Childhood maltreatment and identity diffusion among inpatient adolescents: the role of reflective function. J. Adolesc. 76, 65–74. doi: 10.1016/j.adolescence.2019.08.002, PMID: 31472427

[ref86] Ren X. Wang Y. Xiang H. U. Juan Y. (2019). Social support buffers acute psychological stress in individuals with high interdependent self-construal. Acta Psychol. Sin. 51, 497–506. doi: 10.3724/SP.J.1041.2019.00497

[ref87] Ren D. Wesselmann E. D. Williams K. D. (2013). Interdependent self-construal moderates coping with (but not the initial pain of) ostracism. Asian J. Soc. Psychol. 16, 320–326. doi: 10.1111/ajsp.12037

[ref88] Reyes C. J. (2008). Exploring the relations among the nature of the abuse, perceived parental support, and child's self-concept and trauma symptoms among sexually abused children. J. Child Sex. Abus. 17, 51–70. doi: 10.1080/10538710701884482, PMID: 19842318

[ref89] Ringeisen T. Buchwald P. (2008). “It matters who you turn to: the relational self-construal and communal coping” in Stress and anxiety: Application to life span development and health promotion. eds. Buchwald P. Ringeisen T. Eysenck M. W. (Berlin, Germany: Logos), 75–86.

[ref90] Sakman E. Sümer N. (2022). Testing the compatibility of attachment anxiety and avoidance with cultural self-construals. J. Psychol. 156, 95–116. doi: 10.1080/00223980.2021.20100, PMID: 35015616

[ref91] Santo T. Campbell G. Gisev N. Tran L. T. Colledge S. Di Tanna G. L.. (2021). Prevalence of childhood maltreatment among people with opioid use disorder: a systematic review and meta-analysis. Drug Alcohol Depend. 219:108459. doi: 10.1016/j.drugalcdep.2020.108459 33401031 PMC7855829

[ref92] Scheier M. F. Carver C. S. (1985). The self-consciousness scale: a revised version for use with general populations ^1^. J. Appl. Soc. Psychol. 15, 687–699. doi: 10.1111/j.1559-1816.1985.tb02268.x

[ref93] Sekowski M. Gambin M. Cudo A. Wozniak-Prus M. Penner F. Fonagy P.. (2020). The relations between childhood maltreatment, shame, guilt, depression and suicidal ideation in inpatient adolescents. J. Affect. Disord. 276, 667–677. doi: 10.1016/j.jad.2020.07.056, PMID: 32871699

[ref94] Shahar B. Doron G. Szepsenwol O. (2015). Childhood maltreatment, shame-proneness and self-criticism in social anxiety disorder: a sequential mediational model. Clin. Psychol. Psychother. 22, 570–579. doi: 10.1002/cpp.1918, PMID: 25196782

[ref95] Shams Eldin A. S. Raslan M. R. Madbouly N. M. Enaba D. A. (2019). Anxiety disorders among recovered patients with substance dependence: a follow-up study. Addict. Disord. Treat. 18, 81–88. doi: 10.1097/ADT.0000000000000153

[ref96] Singelis T. M. (1994). The measurement of independent and interdependent self-Construals. Pers. Soc. Psychol. Bull. 20, 580–591. doi: 10.1177/0146167294205014

[ref97] Speidel R. Behrens B. Lawson M. Mark Cummings E. Valentino K. (2023). Latent classes in preschoolers’ internal working models of attachment and emotional security: roles of family risk. Dev. Psychopathol. 35, 1552–1569. doi: 10.1017/S0954579422000293, PMID: 35393923 PMC9547040

[ref98] Suh E. M. Diener E. D. Updegraff J. A. (2008). From culture to priming conditions: self-construal influences on life satisfaction judgments. J. Cross Cult. Psychol. 39, 3–15. doi: 10.1177/0022022107311

[ref100] Tang X. Liu Q. Cai F. Tian H. Shi X. Tang S. (2022). Prevalence of social anxiety disorder and symptoms among Chinese children, adolescents and young adults: a systematic review and meta-analysis. Front. Psychol. 13:792356. doi: 10.3389/fpsyg.2022.792356, PMID: 36072051 PMC9442033

[ref101] Taniguchi H. Kaufman G. (2019). Self-construal, social support, and loneliness in Japan. Appl. Res. Qual. Life 14, 941–960. doi: 10.1007/s11482-018-9636-x

[ref102] Tietjen G. E. Brandes J. L. Peterlin B. L. Eloff A. Dafer R. M. Stein M. R.. (2010). Childhood maltreatment and migraine (part I). Prevalence and adult revictimization: a multicenter headache clinic survey. Headache 50, 20–31. doi: 10.1111/j.1526-4610.2009.01556.x 19845782

[ref103] Torkaman M. Miri S. Farokhzadian J. (2020). Relationship between adaptation and self-esteem in addicted female prisoners in the south east of Iran. Int. J. Adolesc. Med. Health 32:20170168. doi: 10.1515/ijamh-2017-0168 29432204

[ref104] Wang M. Li M. Wu X. Zhou Z. (2022). Cognitive reactivity and emotional dysregulation mediate the relation of paternal and maternal harsh parenting to adolescent social anxiety. Child Abuse Negl. 129:105621. doi: 10.1016/j.chiabu.2022.105621, PMID: 35439628

[ref105] Wang Y. Wang L. (2016). Self-construal and creativity: the moderator effect of self-esteem. Personal. Individ. Differ. 99, 184–189. doi: 10.1016/j.paid.2016.04.086

[ref106] Wang X. D. Wang X. L. Ma H. (1999). Handbook of mental health rating scale, 1st Edn. Beijing: Chinese Mental Health Journal.

[ref107] Wang Y. Yuan Q. Xu Q. (2008). Preliminary application of the self-construal scale (SCS) Chinese version. Chin. J. Clin. Psych. 6, 602–604.

[ref108] Watanabe S. Tarumi Y. Oneschuk D. Lawlor P. Portenoy R. K. Indelicato R. A. (2002). Opioid rotation to methadone: proceed with caution. J. Clin. Oncol. 20, 2409–2410. doi: 10.1200/jco.2002.20.9.2409, PMID: 11981018

[ref109] Watson D. Friend R. (1969). Measurement of social-evaluative anxiety. J. Consult. Clin. Psychol. 33, 448–457. doi: 10.1037/h0027806 5810590

[ref110] Weeks J. W. Heimberg R. G. Rodebaugh T. L. Norton P. J. (2008). Exploring the relationship between fear of positive evaluation and social anxiety. J. Anxiety Disord. 22, 386–400. doi: 10.1016/j.janxdis.2007.04.009 17531437

[ref111] Wei J. Zhang C. Li Y. Xue S. Zhang J. (2015). Psychometric properties of the Chinese version of the fear of negative evaluation scale-brief (BFNE) and the BFNE-straightforward for middle school students. PloS One 10:e0115948. doi: 10.1371/journal.pone.0115948, PMID: 25799570 PMC4370572

[ref112] Wells L. E. (1978). Theories of deviance and the self-concept. Soc. Psychol. 41, 189–204. doi: 10.2307/3033556 366782

[ref1001] WHO (2016). Child Maltreatment. Available at: http://www.who.int/mediacentre/factsheets/fs150/en/ (Accessed November 11, 2017).

[ref113] Wojtynkiewicz E. Sekowski M. (2022). Relations between attachment, identity and borderline personality disorder symptom severity in male inpatients with alcohol use disorder. Personal. Ment. Health 16, 309–318. doi: 10.1002/pmh.1545, PMID: 35475327

[ref114] Wojtynkiewicz E. Sekowski M. Liberacka-Dwojak M. (2021). Differences in the sense of identity between men with alcohol use disorder, drug use disorder, and control group. Psychoanal. Psychol. 38, 223–226. doi: 10.1037/pap0000373

[ref115] Woody S. R. Miao S. Kellman-McFarlane K. (2015). Cultural differences in social anxiety: a meta-analysis of Asian and European heritage samples. Asian Am. J. Psychol. 6, 47–55. doi: 10.1037/a0036548

[ref116] Wu P. Goodwin R. D. Fuller C. Liu X. Comer J. S. Cohen P.. (2010). The relationship between anxiety disorders and substance use among adolescents in the community: specificity and gender differences. J. Youth Adolesc. 39, 177–188. doi: 10.1007/s10964-008-9385-5, PMID: 20084563 PMC2809931

[ref117] Xie D. Leong F. T. L. Feng S. (2008). Culture-specific personality correlates of anxiety among Chinese and Caucasian college students. Asian J. Soc. Psychol. 11, 163–174. doi: 10.1111/j.1467-839x.2008.00253.x

[ref118] Xu J. Ni S. Ran M. Zhang C. (2017). The relationship between parenting styles and adolescents’ social anxiety in migrant families: a study in Guangdong, China. Front. Psychol. 8:626. doi: 10.3389/fpsyg.2017.00626, PMID: 28473798 PMC5397425

[ref119] Yan R. T. (2019). The study of drug addicts' reactions to negative emotional cues and its relationship with impulsivity. Unpublished doctoral dissertation. Guangzhou University.

[ref120] Yeung N. C. Y. Chow T. S. (2019). Coping with my own way: mediating roles of emotional expression and social support seeking in the associations between individual differences and posttraumatic growth. Health Psychol Open 6:205510291984659. doi: 10.1177/2055102919846596, PMID: 31105967 PMC6503603

[ref121] You Z. Zhang Y. Zhang L. Xu Y. Chen X. (2019). How does self-esteem affect mobile phone addiction? The mediating role of social anxiety and interpersonal sensitivity. Psychiatry Res. 271, 526–531. doi: 10.1016/j.psychres.2018.12.040, PMID: 30553099

[ref122] Zhao X. Dang C. Maes J. H. (2020). Effects of working memory training on EEG, cognitive performance, and self-report indices potentially relevant for social anxiety. Biol. Psychol. 150:107840. doi: 10.1016/j.biopsycho.2019.107840, PMID: 31904404

[ref123] Zhao X. F. Zhang Y. L. Li L. F. (2005). Reliability and validity of the Chinese version of the childhood trauma questionnaire. Chin. J. Clin. Rehab. 9, 209–211.

[ref124] Zlomke K. Jeter K. Cook N. (2016). Recalled childhood teasing in relation to adult rejection and evaluation sensitivity. Personal. Individ. Differ. 89, 129–133. doi: 10.1016/j.paid.2015.10.021

